# Application of Latent Class Analysis to Identify Metabolic Syndrome Components Patterns in adults: Tehran Lipid and Glucose study

**DOI:** 10.1038/s41598-018-38095-0

**Published:** 2019-02-07

**Authors:** Noushin Sadat Ahanchi, Farzad Hadaegh, Abbas Alipour, Arash Ghanbarian, Fereidoun Azizi, Davood Khalili

**Affiliations:** 1 Prevention of Metabolic Disorders Research Center, Research Institute for Endocrine Sciences, Shahid Beheshti University of Medical Sciences, Tehran, Iran; 2 Department of Epidemiology, School of Public Health, Shahid Beheshti University of Medical Sciences, Tehran, Iran; 3 Safety Promotions and Injury Prevention Research Center, Shahid Beheshti University of Medical Sciences, Tehran, Iran; 4 Endocrine Research Center, Research Institute for Endocrine Sciences, Shahid Beheshti University of Medical Sciences, Tehran, Iran; 5 Department of Biostatistics and Epidemiology, Research Institute for Endocrine Sciences, Shahid Beheshti University of Medical Sciences, Tehran, Iran

## Abstract

In this study, using latent class analysis (LCA), we investigated whether there are any homogeneous subclasses of individuals exhibiting different profiles of metabolic syndrome (MetS) components. The current study was conducted within the framework of the Tehran Lipid and Glucose Study (TLGS), a population-based cohort including 6448 subjects, aged 20–50 years. We carried out a LCA on MetS components and assessed the association of some demographic and behavioral variables with membership of latent subclasses using multinomial logistic regression. Four latent classes were identified:(1) Low riskclass, with the lowest probabilities for all MetS components (its prevalence rate in men: 29%, women: 64.7%), (2) MetS with diabetes medication (men: 1%, women: 2.3%), (3) Mets without diabetes medication (men: 32%, women: 13.4%), (4) dyslipidemia (men: 38%, women: 19.6%). In men the forth subclass was more significantly associated with being smoker (odds ratio: 4.49; 95% CI: 1.89–9.97). Our study showed that subjects with MetS could be classified in sub-classes with different origins for their metabolic disorders including drug treated diabetes, those with central obesity and dyslipidemia associated with smoking.

## Introduction

Metabolic syndrome (MetS) is characterized by a clustering of various interrelated risk factors including abdominal obesity, dyslipidemia, low-HDL-cholesterol, hyper-triglyceridemia and high blood pressure; which together culminate in adverse consequences, including type 2 diabetes and cardiovascular disease (CVD). Concerns have recently been raised about the validity of diagnosis of MetS using current standard cut offs^1,2^. Examples of some of these uncertainties are: (1) Unknown causes of Mets while it has been originally hypothesized that insulin resistance is the underlying pathology influencing all MetS components; research has shown that not all individuals meeting criteria of MetS are insulin resistant (2) lack of experimental evidences for the cutoff points of components for MetS diagnosis; proposed MetS criteria specify different cut points for men and women regarding waist circumference and HDL cholesterol, suggesting that the risk conferred by some MetS components differs by gender, (3) considering different numbers of MetS components as a risk continuum was a more valuable risk predictor than identifying MetS, (4) unknown subclasses of MetS. Several statistical ways including factor analysis have been used in order to find underlying biology of MetS^3,4^. Considering the possibility that relationship among MetS components may meaningfully differ in different subclasses, LCA can identify distinct profiles of persons based on their presentation of MetS components. So, it has a person-centered approach which is concerned with the structure of groups (or cases), as oppose to factor analysis which is variable-centered i.e. it a works with correlation between variables. On the other hand, LCA includes discrete latent categorical variables with multinomial distribution, but factor analysis includes continuous latent variables with the assumption of normal distribution^5^.

Cluster analysis, in comparison to LCA has less robust empirically-based indices for class number decisions and quality of class solutions^6^. Prior methods to identify the underlying features of the MetS may not be usable foraddressing the question of whether a condition that is present or absent explains the observed manifestations of metabolic syndrome^7^. LCA aims to classify similar individuals into groups (or latent clusters), in which each latent cluster is viewed as consisting of homogeneous individuals with regards to the observed variables (in our case, components of MetS), and the different latent clusters are viewed as representing the unobserved heterogeneity among individuals in these observed variables^8^. This study aimed to examine: 1) whether there are any homogeneous subclasses of individuals exhibiting different profiles of MetS components and 2) whether there is an association between these subclasses and individual characteristics like age, education, smoking and physical activity.

## Result

Men (n = 2597) had a mean (SD) age of 34.2(8.2) years and 32% of them were smokers; corresponding values were 33.9(8.40) and 4.2% in women (n = 3851). Proportions of men and women with each metabolic syndrome component are shown in Table 1. The results show that except HDL, the prevalence of all MetS components was more common in men than women.

**Table 1:** Sample characteristics (N = 6448).

Variables	Men (n = 2597) Mean (SD) or proportion	Women (n = 3851) Mean (SD) or proportion	P value
**General characteristics**			
Age (years)	34.2 (8.2)	33.9 (8.40)	0.130
Smoking status (current) (%)	32	4.2	0.001
Education (greater than diploma) (%)	21	11.7	<0.0001
Marital status (single) (%)	27.1	16.6	<0.0001
Low Physical activity (%)	78	72.7	<0.0001
Glucose (mmol/dl)	5.13 (1.11)	5.08 (1.33)	0.007
Systolic blood pressure (mm Hg)	114.7 (12.7)	112.2 (13.6)	<0.0001
Diastolic blood pressure (mm Hg)	76.2 (10.07)	75.9 (9.6)	<0.0001
HDL cholesterol (mmol/dl)	0.98 (0.23)	1.15 (0.28)	<0.0001
Triglycerides (mmol/dl)	1.70 (1.14–2.51)*	1.31 (0.91–1.97)*	<0.0001
Waist circumference (cm)	86.6 (11.3)	84.5 (12.3)	<0.0001
**Binary variables related to metabolic syndrome**			
High glucose (%)	15.4	14	0.110
High triglyceride (%)	50.4	34.3	<0.0001
High waist (%)	24.2	21.3	0.007
LowHDL (%)	65.6	75	<0.0001
High blood pressure (%)	23.2	19.6	<0.0001
Antihypertensive medication (%)	0.8	2.7	<0.0001
Lipid-lowering medication (%)	1	1.4	<0.0001
Diabetes medication (%)	1	1.5	<0.0001
Mets	40.0	28.2	<0.0001

*Expressed as median (interquartile range).

### Number of latent clusters

A four-class basic model considered as optimal had the highest entropy of 0.74 with a better interpretation compared to others (Supplementary Table 1). Table 2 shows the conditional probabilities of a “Yes” response (item response probabilities) to MetS components. These probabilities form the basis for interpretation and labeling of the latent classes. The greater probabilities appear in bold font, to highlight the overall pattern. The largest class (low risk class) with the prevalence of 60.6% showed the lowest probabilities for all metabolic syndrome components and characterized by individuals who were healthy regarding MetS components except for HDL. The second latent class, (MetS with diabetes medication) with a prevalence of 1.3% was the smallest one with high probability for high glucose component (0.97) and use of glucose-lowering medications (1.00). The third latent class (MetS without diabetes medication) with prevalence of 13.7% was distinguished by high prevalence of MetS components (all item responses ≥ 0.5) except high glucose with an item response of 0.4. Individuals in this class had no medication for diabetes. The fourthclass (dyslipidemia), with a prevalence of 24.4%, was distinguished by higher probabilities of high TG (1.00) and low-HDL (0.91) (Table 2).

**Table 2:** Estimated prevalence of latent classes and the probability of observed metabolic syndrome components for each subclass.

	Low risk	MetS with diabetes medication	MetS without diabetes medication	Dyslipidemia
**Latent class prevalence**	**60.6%**	**1.3%**	**13.7%**	**24.4%**
**Item-response probabilities of metabolic syndromecomponents**				
high glucose	0.06	**0.97**	0.41	0.16
High waist	0.09	0.47	**0.65**	0.29
low HDL	0.61	**0.83**	**0.78**	**0.91**
High triglyceride	0.09	**0.70**	**0.70**	**1.00**
High blood pressure	0.11	0.46	**0.64**	0.22
Lipid-lowering medication	0.00	0.17	0.04	0.01
Diabetes medication	0.00	**1.00**	0.00	0.00
Antihypertensive medication	0.00	0.19	0.08	0.006

### Sex differences in subclass membership

Sex was entered as a grouping variable in the LCA to establish whether the item-response probabilities are invariant across the two genders. A four-class solution was found optimal for both genders and the pattern of item response probabilities suggested identical latent class labels for males and females. We also assessed whether the prevalence of subclasses was the same or different across genders and found that overall difference in the prevalence was highly significant (p < 0.001) (Table 3). Characteristics of individuals and the proportion of the MetS components were reported for each subclass in both men and women (Table 4).

**Table 3:** Estimated prevalence of latent classes and the probability of observed metabolic syndrome components for each subclass in men and women.

	C1 (Low risk)	C2 (MetS with diabetes medication)	C3 (MetS without diabetes medication)	C4 (Dyslipidemia)
**Latent class prevalence**				
Men (%)	**29**	**1.0**	**32**	**38.1**
Women (%)	**64.7**	**2.3**	**13.4**	**19.5**
**Item-response probabilities of metabolic syndrome components**				
Men				
high glucose	**0.10**	**1.00**	0.29	0.05
High waist	**0.10**	0.44	**0.54**	0.09
low HDL	**0.00**	**0.76**	**0.83**	**1.00**
High triglyceride	**0.19**	**0.60**	**0.87**	0.42
High blood pressure	**0.18**	0.36	0.41	0.10
Lipid-lowering medication	**0.00**	0.12	0.02	0.00
Diabetes medication	**0.00**	**1.00**	0.00	0.00
Antihypertensive medication	**0.00**	0.04	0.01	0.00
Women				
High glucose	**0.05**	**0.96**	0.39	0.16
High waist	**0.08**	**0.51**	**0.65**	0.29
LowHDL	**0.66**	**0.88**	**0.79**	**0.98**
High triglyceride	**0.08**	**0.76**	**0.60**	**0.97**
High blood pressure	**0.09**	**0.51**	**0.57**	0.23
Lipid-lowering medication	**0.00**	0.20	0.02	0.02
Diabetes medication	**0.00**	**0.66**	0.00	0.00
Antihypertensive medication	**0.008**	0.25	0.10	0.01

**Table 4:** The characteristics of individuals and the proportion of the MetS components in different subclasses.

Men	Low risk (n = 799)	MetS with diabetes medication (n = 25)	MetS without diabetes medication (n = 738)	Dyslipidemia (n = 1035)	P value
Age (years)	32.55(8.41)	42.76(7.40)	37.12(7.48)	33.22(8.09)	<0.0001
Smoking status (Current), n (%)	26.8	32.0	33.3	35.1	<0.0001
High glucose	10.9	100	34.8	3.0	<0.0001
High waist	9.5	44.0	65.4	5.6	<0.0001
Low HDL	0.0	76.0	87.9	100	<0.0001
High triglyceride	21.5	60.0	94.3	41.3	<0.0001
High blood pressure	20.2	36.0	50.0	6.1	<0.0001
Antihypertensive medication use (%)	0.1	4.0	2.0	3.0	<0.0001
Lipid-lowering medication use (%)	0.1	12.0	2.8	2.0	<0.0001
Glucose-lowering medication use (%)	0.0	100	0.0	0.0	<0.0001
Mets	16.0	0.68	98.8	15.9	<0.0001
Insulin resistance (%)*	9.0	70.0	43.0	11.3	<0.0001
**Women**	**Low risk (n = 2406)**	**MetS with diabetes medication (n = 66)**	**MetS without diabetes medication (n = 421)**	**Dyslipidemia (n = 958)**	**P value**
Age (years)	31.48 (7.96)	42.93 (4.74)	40.22 (6.69)	36.75 (7.54)	<0.0001
Smoking status (Current), n (%)	3.9	9.1	3.8	4.8	<0.0001
High glucose	4.7	97.2	42.8	18.8	<0.0001
High waist	8.1	51.5	82.9	25.4	<0.0001
Low HDL	65.4	87.9	70.8	100	<0.0001
High triglyceride	2.4	75.8	60.1	100	<0.0001
High blood pressure	8.6	50.0	79.3	18.8	<0.0001
Antihypertensive medication use (%)	7.0	27.3	14.7	8.0	<0.0001
Lipid-lowering medication use (%)	1.0	28.2	3.1	1.9	<0.0001
Glucose-lowering medication use (%)	0.0	89.4	0.0	0.0	<0.0001
Mets	2.0	93.9	82.8	30.2	<0.0001
Insulin resistance (%)*	11.9	80.7	43.6	31.9	<0.0001

*We considered a HOMA-IR more than 2.38 in men and 2.68 in women as indicating the presence of Insulin resistance. The number of participants with available insulin data was 2785 (1749 men).

### Association of covariates with latent cluster membership

A multinomial logistic regression analysis was conducted to identify predictors associated with metabolic syndrome class membership using latent class 1, low risk class, as the reference; this showed that higher age was associated with significantly higher odds of belonging to the other classes versus latent class 1 in both genders. Men in latent class 3 were more likely to have low physical activity and smoking experience than those in the latent class 1. Being a current smoker was associated with significantly higher odds of belonging to the latent class 4 compared to latent class 1 (OR = 4.49 for men). Compared to the latent class 1, women in the latent class 2 were more likely to be current smoker (Table 5). We did additional analysis onlyonparticipants with available insulin data (n = 2785) and showed that increasing HOMA-IR was significantly associated with both MetS subclasses (Table 6). However, considering latent class 2 (MetS with diabetes medication) as the reference group showed that with 1 SD increase in HOMA-IR, the odds for being classified into class 3 (MetS without diabetes medication) was 23% lower than the odds for being classified into class 2 (MetS with diabetes medication) in men; the corresponding value was 19% in women (OR = 0.67, 95% CI 0.52–0.88 in men and 0.81, 0.65–1.01 in women) (Table 6).

**Table 5:** Associations between latent class membership with demographic and lifestyle variables.

Predictors	Low risk (REF.)	MetS with diabetes medication Odds (95% CI)	MetS without diabetes medication Odds (95% CI)	Dyslipidemia Odds (95% CI)
**Men**	**n = 799**	**n = 25**	**n = 738**	**n = 1035**
Age (year)	REF	1.26 (1.19–1.32)*	1.20 (1.14–1.25)*	1.11 (1.07–1.15)*
Education (diploma and higher)	REF.	0.509 (0.15–1.64)	1.059 (0.77–1.43)	0.942 (0.72–1.21)
Low Physical activity	REF.	0.96 (0.38–2.46)	1.42 (1.04–1.94)*	0.95 (0.74–1.22)
Smoking status (Current)	REF.	2.270 (0.74–6.68)	2.30 (0.97–5.44)	4.49 (1.89–9.97)*
**Women**	**n = 2406**	**n = 66**	**n = 421**	**n = 958**
age	REF.	1.40 (1.33–1.46)*	1.33 (1.23–1.43)*	1.17 (1.13–1.20)*
Education (diploma and higher)	REF.	0.306 (0.04–2.29)	0.150 (0.02–1.09)	0.430 (0.20–0.91)*
Low Physical activity	REF.	0.67 (0.39–1.16)	1.19 (0.80–1.7)	1.15 (0.87–1.50)
Smoking status (Current)	REF.	2.688 (1.08–6.61)*	0.539 (0.27–10.4)	1.332 (0.77–2.29)

**Table 6:** Associations between latent class memberships with HOMA_IR variable.

Predictor	Low risk (REF.)	MS with diabetes medication Odds (95% CI)	MS without diabetes medication Odds (95% CI)	Dyslipidemia Odds (95% CI)
**Men**				
HOMA_IR	REF	2.35 (1.98–2.80)	2.06 (1.81–2.33)	1.79 (1.61–1.99)
HOMA_IR	0.13(0.08–0.20)	REF	0.67 (0.52–0.88)	0.17 (0.12–0.25)
**Women**				
HOMA_IR	REF	3.71 (2.82–4.86)	2.88 (2.32–3.56)	1.19 (0.97–1.46
HOMA_IR	0.26 (0.20–0.34)	REF	0.81 (0.65–1.01)	0.65 (0.51–0.83)

## Discussion

The person-centered analytic approach of LCA sought to improve the comprehension of metabolic syndrome, by identifying latent classes based on MetS component patterns. Considering clustering of MetS components can be an effective approach in preventive strategies. Some studies emphasized the clustering and combining patterns of MetS components^7,9^. LCA revealed four distinct subclasses of MetS among an Iranian population, aged 20–50 years: (1) low risk, (2) MetS with diabetes medication, (3) MetS without diabetes medication, (4) dyslipidemia.

Clinicians believe that risk factors of cardiovascular disease tend to accumulate, and thus the risk of developing cardiovascular disease increases along with increments in their clustering abilities^10,11^. Findings of our study demonstrate that two latent classes (the second and third) were consistently associated with many features of Mets; these latent classes may demonstrate a specific clinical situation generating the observed features of MetS. Our results indicate this clustering of MetS components in two different patterns. The second latent class was more relevant to the insulin resistance.

Longitudinal studies have shown that there are different risks of mortality following the different combination of MetScomponents^12,13^; our results showed different combination pattern of Mets components as well. For example, in the combination of MetS components among individuals of latent class 3, we found out that the high glucose component haslimited role in the concept of MetS. Based on these patterns, the individuals of latent classes 2 and 3 probably have different chance of developing complications of metabolic syndrome.

The prevalence of each subclass differed between men and women, although both genders had the same number of latent classes and the same item response probabilities. Age, education, low physical activity and smoking status were somehow correlated with MetS latent class membership. The LCA method produces estimates of conditional probabilities indicating the association between the observed measures and latent classes. In our analysis, latent class 1, the largest group, characterized by very low association with all MetS components. Latent class 2 was strongly associated with high glucose and glucose-lowering medications and was also related to the other Mets components, possiblyrepresenting a pathophysiologic state producing the observed components of the Mets^7^. Since insulin resistance is supposed to be the core of MetS^14–16^, the latent class 2 could be a good surrogate forthisdefinition. The latent class 3 was also related to all MetS components with response item probability >0.5 except for the high blood glucose component; additional analysis showed that the association of insulin resistance with this class is lower than that with latent class 2. None of individuals in this subclass used diabetes medication, a finding contrary to the insulin resistance hypothesis for MetS, which may show that some people with MetS have other origins for their MetS components^17,18^. Obesity can be a good explanation for developing MetS in latent class 3 because body fat, not insulin resistance, can be an inflammatory cardio metabolic risk marker. In our study, individuals in the MetS without diabetes medication subclass were more obese than other groups, with a response item probability of 0.65 for the high waist component.

Latent class 4 was associated mainly with low HDL and elevated triglycerides, but had a weak association with other Mets components. We know that low HDL and high TG are metabolically linked and TG/HDL is an independent risk factor for cardiovascular diseases^19^. Since smoking has a strong association with latent class 4, it seems that this subclass mostly includes smokers, with a low prevalence of obesity, compared to other high risk sub groups (latent classes 2 and 3); there was stronger association between this group and smoking (OR = 4.5) compared to others (OR = 2.3) in our male population. Previous studies have confirmed the association between smoking and dyslipidemia^20,21^. It needs to be mentioned that the prevalence of smoking was very low in our female population.

Sex differences in the prevalence of latent class 3 were striking, with almost twice as many men in this subclass. Results indicated higher proportion of women (64.4%) classified into the latent class 1 compared to men (29%). Latent class 1 was the most prevalent subclass in women but was the ranked third among men. The differences in the subclass prevalence between women and men is consistent with men having a higher risk of MetS than women, a finding contrary to national MetS prevalence estimates, with higher prevalence of MetS in women (32.9%) compared to men (22.9%) and European data, i.e. 10% in men and 13% in women^22^. Although previous studies in the TLGS confirm the prevalence of MetS as being higher in females^23,24^, the difference between their results and ours could be due to the age distribution. Since mean age for menopause is 50 years in Iranian women^25^, most of our female participants were not menopausal.

MetS subclasses were identified related to the covariates in a manner consistent with previous researches. Smokers had higher odds of being classified into latent class 2, latent class 3 and latent class 4 compared to those in latent class 1, with high significance for latent class 4 in men and latent class 2 in women. These results are consistent with those reported by Yankey *et al*. who found that smoking was significantly associated with increased odds of hypertension and hyperglycemia after adjusting for confounders^26^. Other studies have also reported a strong association between smoking and dyslipidemia^20,21,27^. Both men and women with low physical activity were more likely to be in the latent class 3 than in the latent class 1. Meta-analyses of prospective cohort studies conducted on. individuals from different populations reported that a high level of leisure time physical activity was statistically associated with decreased risk of the MetS^28^; findings also reported in another short term study conducted among Korean male workers^29^. Older men and women weremore likely to belong to the three classes than latent class 1; the odds favoring metabolic syndrome significantly increased with age, and decreased with education level^30^. Twoprevious studies showed a higher prevalence of MetS in elderly subjects and a larger number of component clustering of the syndrome in elderly patients, compared with younger patients^31,32^.

Overall, clustering of components of MetS can be helpful and valuable in reinforcing the hypothesis that different pathophysiological mechanisms are engaged in this process. The most important finding in our study is that among individuals of latent classes of 2 and 3, some of the Mets components did not play an influential role (e.g. of latent class 2 “high blood pressure” and “high waist”; of latent class3 “high glucose”). This results are consistent with previous studies^33,34^.

Women in low risk group had a very high prevalence of low HDL (65%), while the prevalence of other MetS components was very low in women. Previous studies showed that the low HDL cholesterol is the most common metabolic abnormality in the Middle East including Iran, especially in women^35,36^. Among Iranian women aged 25–64 years, the prevalence of low HDL cholesterol is 84%^36^ which is so different from western countries^37^. The amount of low HDL cholesterol is low even in people without metabolic syndrome^38^. In our study the low risk group had the lowest prevalence of low HDL cholesterolamong all subclasses.

The most important short comings of LCA are that it can detect an unobserved structure only if each of its classes is large enough to be detected. Detecting rare latent classes would be difficult when sample size decreases. Another limitation of LCA is due to the temptation to attach too much meaning to a latent class and reification of the labels assigned to each class for easier interpretation^39^. As another limitation in our study, changing cut off points for defining MetS components may results in different LCA outputs. Furthermore, behavioral changes among people over time may also change the subclasses identification. Considering young and midlife adults, before menopause age, allows the results to be comparable between both genders. On the other hand, we selected this age range to avoid the effect of co-morbidities and medications on the results. Although this age range increases the internal validity of the results, it may decrease their external validity. Our study has several strengths. (1) Using a new approach to classify high risk people based on their observed cardio metabolic risk factors. (2) We used a large sample size. (3) Utilizing community-based data from the TLGS, although this sample is not nationally representative and inferences cannot be extended to the other communities especially rural populations.

## Conclusion

Using LCA showed that subjects with MetS could be classified in sub-classes with different origins for their metabolic disorders including drug treated diabetes, those with central obesity and dyslipidemia associated with smoking.

## Methods

### Sample

This study was conducted within the framework of the Tehran Lipid and Glucose Study (TLGS) whichis a large-scale population-based prospective cohort study with long-term follow-up, performed on a representative sample of residents of Tehran. The baseline measurements (phase 1) were performed between 1999 to 2001^40^. For the current study, subjects aged 20–50 years were selected from the first phase (n = 6974); this range of age was used to avoid the effect of co-morbidities and menopause on our results. From these, we excluded 526 participants with missing data on MetS components, resulting in 6448 subjects. Informed written consent was obtained from all participants. The study was approved by the ethics committee of the Research Institute for Endocrine Sciences. All methods of the current study were performed in accordance with the relevant guidelines and regulations.

### Measures

#### Measurement and definition of terms

Data were collected using interviews, physical examinations and laboratory measurements. Details of measurement of MetS components, including waist circumference (WC), fasting plasma glucose (FPG), systolic and diastolic blood pressure (SBP and DBP, respectively), high-density lipoprotein cholesterol (HDL-C), and triglycerides (TG), have been described elsewhere (12). Components of MetS were: Elevated waist circumference (≥95 cm for both Iranian men and women), elevated blood pressure (systolic/diastolic blood pressure ≥85/130 mm Hg), low HDL- cholesterol (<40 mg/dlin men and <50 mg/dlin women), elevated triglycerides (high TG) (≥150 mg/dl), and high glucose (≥100 mg/dl)^41^. Drug treatments including anti-hypertensive drugs, lipid medication and diabetes medication were considered as separate binary variables. Fasting serum insulin was analyzed by the electro chemiluminescence immunoassay method 42 and HOMA-IR calculated as: FPG (mmol/L) × fasting serum insulin (μU/mL)/22.5. We considered HOMA-IR more than 2.38 in men and 2.68 in women as indicating the presence of insulin resistance^42^. The number of participants with available insulin data was 2785 (1749 men).

#### Variables considered for latent class analysis

Eight dichotomous observable variables, including high glucose, low HDL, high WC, high TG, high blood pressure and drug treatments including anti hypertension, lipid medication and diabetes medication were considered forthe latent class analysis. All definitions of variables are based on the cut points defined for the metabolic syndrome definition^43,44^.

#### Consideration of sex in the LCA

In previous studies the prevalence of MetS differed by gender^4^. We evaluated: (1) whether men and women experienced the same number and prevalence of MetS subclasses. (2) Whether the item response probabilities differ across genders.

#### Measurement of covariates

We considered demographic and lifestyle variables as correlates of the MetS subclass membership: Age (years), smoking (current vs. never or past smoker), education (categorized as ≤diploma and >diploma, with ≤diploma serving as the reference group); physical activity (those with ≥3 times a week moderate to high intensity physical activity vs. those with less than 3 times a week physical activity), marital status (married vs. single, widowed, or divorced). We did an additional analysis only for participants who had insulin data (n = 2785).

### Statistical analysis

There were three analytic phases. (1) Explaining the sample and developing the LCA model. (2) Evaluating sex differences in MetS subclasses. (3) Evaluating the association between covariates and MetS subclasses.

First, summary statistics were separated by sex. We then constructed LCA models with numbers of subclasses varying from two to six. Basic models without grouping variables and covariates were fitted first to obtain a general understanding of the structure of the subclasses. These models were examined for fit, parsimony and interpretability of the observed variables. Akaike Information Criterion (AIC), Bayesian Information Criterion (BIC), entropy, sample size Adjusted Bayesian Information Criteria (ABIC)^45^, adjusted LMR LR (ALMR LR) test, and interpretability of competing solutions were considered to select the model with the optimal number of MetS latent classes. Larger entropy and lower AIC and BIC values indicated better fitting solution. A significant P-value of the LMR LR test (P < 0.05) indicates a significant improvement in this model fit in the k-class model compared with the (k-1) class model^46^.

Second, optimal number of latent classes was selected for the basic model and the model was stratified by sex as a grouping variable to see whether the number of latent subclasses was identical between men and women. We found that the number of latent subclasses and item –response probabilities were identical between men and women. However, the prevalence of sub types was different across genders. As a result, measurement invariance doesn’t hold across genders for the prevalence of sub types^45^.

Finally, we evaluated the association between subclasses of MetS and demographic or lifestyle covariates. Low risk class served as the reference group to facilitate interpretation. Odds ratios were calculated to show the odds of being in each class, compared to being in the reference. Analyses were conducted using LCA Stata Plugin Users’ Guide Version 1.2.1 and Mplus version 5.

## Supplementary information

supplementory info
