# Supplementary material for: Application of Latent Class Analysis to Identify Metabolic Syndrome Components Patterns in adults: Tehran Lipid and Glucose study
Source: Sci Rep. 2019 Feb 7;9:1572. doi: 10.1038/s41598-018-38095-0 (PMC6367385; doi:10.1038/s41598-018-38095-0)
Supplement: Supplementary file 1 — supplementory info [file 41598_2018_38095_MOESM1_ESM.docx]

**Application of Latent Class Analysis to Identify Metabolic Syndrome Components Patterns in adults: Tehran Lipid and Glucose study**

**Noushin Sadat Ahanchi^1,2^, Farzad Hadaegh^2^, Abbas Alipour^3^,Arash Ghanbarian^2^,Fereidoun Azizi^4^,DavoodKhalili^2,5,*^**

***^1^Department of Epidemiology, School of Public Health, ShahidBeheshti University of Medical Sciences, Tehran, Iran***

***^2^Prevention of Metabolic Disorders Research Center, Research Institute for Endocrine Sciences, ShahidBeheshti University of Medical Sciences, Tehran, Iran***

3**Safety Promotions and Injury Prevention Research Center, ShahidBeheshti University of Medical Sciences,**

**Tehran, Iran.**

***^4^Endocrine Research Center, Research Institute for Endocrine Sciences, ShahidBeheshti University of Medical Sciences, Tehran, Iran***

***^5^Department of Biostatistics and Epidemiology, Research Institute for Endocrine Sciences, ShahidBeheshti University of Medical Sciences, Tehran, Iran***

**^*^ Corresponding author:**

**DavoodKhalili**

**P.O. Box: 19395-4763, Tehran, Iran**

**Phone: +982122432500**

**Fax: +982122416264**

**E-mail: d**[**khalili@endocrine.ac.ir**](mailto:khalili@endocrine.ac.ir)

| **Supplementary Table 1. Model fit information for competing latent class models (N=6448).** | | | | | | | |
| --- | --- | --- | --- | --- | --- | --- | --- |
| Number of subclasses | Number of parameters | df | AIC | BIC | ABIC | Entropy | ALMR LR  test P-value |
| Two | 17 | 237 | 36634.7 | 36749.8 | 36695.8 | 0.55 | 0.000 |
| Three | 26 | 229 | 36363.4 | 36539.5 | 36456.8 | 0.66 | 0.000 |
| Four* | 35 | 220 | 36267.0 | 36504.1 | 36392.92 | 0.74 | 0.001 |
| Five | 44 | 211 | 36230.3 | 36528.3 | 36388.5 | 0.70 | 0.0185 |
| Six | 53 | 202 | 36211.9 | 36570.8 | 36402.4 | 0.69 | 0.207 |
| Note. Df =degrees of freedom; AIC= Akiake Information Criterion; BIC=Bayesian Information  Criterion; ABIC, sample size adjusted Bayesian information criteria  * Selected as final model | | | | | | | |
